# Supplementary material for: NewtCap: An Efficient Target Capture Approach to Boost Genomic Studies in Salamandridae (True Salamanders and Newts)
Source: Ecol Evol. 2025 Aug 12;15(8):e71835. doi: 10.1002/ece3.71835 (PMC12343749; doi:10.1002/ece3.71835)
Supplement: Supplementary file 1 — Data S1: ece371835‐sup‐0001‐SupinfoS1.zip. [file ECE3-15-e71835-s001.zip › Protocol - C0t-1 DNA for Triturus Newts V1.0.docx]

Protocol: C_0_t-1 DNA for *Triturus* Newts

V 1.0 – James France

Based on the protocol from the UCLA Shaffer Lab.

**Reagents required:**

- 1 × and 0.1 × TE buffer
- S1 Nuclease & S1 Nuclease 5x Buffer (we order these from ThermoFisher: code EN0321)
- 3M Sodium Acetate solution
- Isopropanol (Ice cold)
- 70-80 % ethanol (Ice cold)

**Equipment required:**

- 200 µl and 1.5 ml microcentrifuge tubes
- 10 μl, 20 μl, 200 µl and 1 ml pipettes and tips
- Cool blocks for microcentrifuge tubes
- Vortexer and tube spinner
- Thermocycler
- Heat block
- Microcentrifuge
- Vacuum centrifuge / concentrator
- Covaris or other equipment for shearing DNA
- Nanodrop or other equipment for quantifying DNA

**Notes:**

C_0_t-1 DNA is enriched in repetitive sequences, which makes it useful as a block in target capture. C_0_t-1 DNA is prepared by first denaturing sheared, genomic DNA and then allowing the DNA to re-anneal for a brief period of time. During this time repetitive sequences are far more likely to successfully anneal than unique sequences (as their effective concentration is vastly higher). This results in a mixture of double stranded repetitive DNA and single stranded DNA with unique sequences. By selectively digesting the single stranded DNA, we are left with only repetitive sequences.

C_0_t-1 derives from C_0_ × t = 1, where **C_0_** is the starting concentration of DNA *nucleotides* and **t** is the time for re-annealing (and **1** is 1 mol/L × s). Thus, one can calculate a C_0_t-1 re-annealing time for any initial concentration of DNA. For example, assuming a weight per nucleotide of 339 g/mol, DNA at 1,000 ng/µl has a concentration of 2.94 **m**mol/L. Then: $\frac{1 mol/L \times s}{2.94 \times{10}^{-3} mol/L}=339 s=5\min39 sec$.

Ideally, for Salamanders at least, a good yield of C_0_t-1 DNA is 30-60 % of the starting DNA (according to the UCLA Shaffer Lab). If the yield is too low, we may not be including significant repetitive sequences you want to block (as well as wasting DNA), and if the yield is too high then we may be blocking unique sequences. We found that our calculated re-annealing time gave insufficient yields in *Triturus* so we increased the re-annealing time to 7 min 30 seconds. This gives a yield of around 35%.

C_0_t-1 DNA seems effective for relatively divergent species, our *Triturus* C_0_t-1 works perfectly for *Ommatotriton,* for example. Initial results suggest our target capture protocol using Triturus C_0_t-1 DNA is useful throughout Salamandridae.

Other taxa (than Salamanders) have vastly different proportions of repetitive DNA, so the target yield and time point probably require optimization in each case. When optimizing have found it is easier to keep a consistent starting DNA concentration of 1 µg/µl and adjust the re-annealing time.

A **lot** of starting DNA is required. With a yield of 35%, **1 mg** of starting DNA gives 350 µg of C_0_t-1 DNA. Since we use 30 µg per capture reaction, that’s less a dozen pools. Get plenty of fresh tissue.

Depending on your equipment, shearing the DNA can be a problem. Our Covaris sonicator can only hold a single 50 µl tube, so processing 500 µl gets tedious. However, we have found that the shearing quality at 1,000 ng/µl remains excellent, despite the recommendations of the manufacturer’s manual.

Measuring higher concentrations of DNA can be problematic, depending on your equipment. You may have to dilute to get accurate readings.

This protocol is based around batches of 500 µg input DNA, but is easily scaled up or down. In practice we typically make C_0_t-1 DNA in batches of 1 µg or more.

**Procedure:**

1. Extract high molecular weight DNA from tissue of *Triturus* (or another target taxon). Elute the extracted DNA in 1 × TE buffer. Aim for a concentration of at least 1000 ng/µl. (In our lab we use a simple salting out extraction using the Promega Wizard Genomic DNA Purification Kit)
2. Measure concentration of DNA extracts (via the Nanodrop or equivalent instrument)
3. Pool sufficient extracts for 500 **µg** of total DNA and adjust volume to 500 µl with 1 × TE buffer (so final DNA concentration to 1,000 ng/µl)

1. Shear DNA to 200-500 bp in size (via Covaris or equivalent instrument, depending on your equipment this may have to be performed in multiple batches)
2. Split sheared DNA across 50 μl aliquots in 200 µl PCR tubes. Place in the Thermocycler and run the following program:

| Temperature | Time |
| --- | --- |
| 95 °C | 5 min |
| 60 °C | 7 min 30 sec* |
| 4 °C | ∞ |

**Or whatever your calculated and optimized C_0_t-1 re-annealing time is*

1. After the thermocycler has reached 4 °C, remove the tubes and place on ice for 2 minutes. Recombine the aliquots into 500 μl.
2. Prepare the S1 Nuclease solution:

| Component | μl (for 500 μl DNA) |
| --- | --- |
| Ultra-pure water | 99.5 |
| S1 Nuclease | 0.5 |
| S1 Nuclease Buffer | 150 |
| Total | **250** |

1. Transfer the DNA to a 42 °C heat block and preheat the S1 nuclease solution to 42 °C
2. Add 250 μl S1 Nuclease solution to the DNA (mix well)
3. Incubate for 1 h at 42 °C
4. Add 75 µl 3M sodium acetate and 750 µl ice cold isopropanol, mix gently
5. Centrifuge at 14,000 rpm for 20 min (if possible, keep at 4 °C)
6. Remove isopropanol from tube, being careful not to disturb the pellet
7. Add 500 µl ice-cold 70-80 % ethanol and gently wash the pellet
8. Centrifuge at 14,000 rpm for 10 min (if possible, keep at 4 °C)
9. Remove ethanol from tube, being careful not to disturb the pellet
10. Let the pellet dry
11. Rehydrate in 500 µl 0.1 × TE buffer and measure concentration (via the Nanodrop or equivalent instrument)
12. Vacuum concentrate to 6,000 ng/µl and store finished C_0_t-1 DNA at -20 °C
