# Supplementary material for: NewtCap: An Efficient Target Capture Approach to Boost Genomic Studies in Salamandridae (True Salamanders and Newts)
Source: Ecol Evol. 2025 Aug 12;15(8):e71835. doi: 10.1002/ece3.71835 (PMC12343749; doi:10.1002/ece3.71835)
Supplement: Supplementary file 1 — Data S1: ece371835‐sup‐0001‐SupinfoS1.zip. [file ECE3-15-e71835-s001.zip › Protocol - Library Prep with Enzymatic Fragmentation v1.1.docx]

Protocol: Library Preparation with Enzymatic Fragmentation (Quarter Volume)

V 1.1n – James France

Based on the manual for the NEBNext® Ultra™ II FS DNA Library Prep Kit (Version 2.2_6/20)

**Reagents required:**

- NEBNext® Ultra™ II FS DNA Library Prep Kit for Illumina – New England Bioscience E7805L
- NEB adaptors and USER enzyme (Naturalis buys these – in NGS lab freezer drawer 3)
- Index primers for Illumina sequencing (we have a plate of these for in our NGS freezer drawer)
- Magnetic separation beads (Naturalis buys NucleoMag beads from Macherey Nagel – these are available in the NGS fridge in 1.5 ml tubes labeled MN)
- Freshly prepared 70% ethanol
- 0.1 x TE buffer (the library prep kit comes with 1 x TE buffer, you can dilute this down with nuclease free water)

**Equipment required:**

- Thermocycler (ideally TC-10 or TC-12 as they take skirted plates)
- Magnetic separation block for skirted plates
- Cool blocks for PCR plates and microcentrifuge tubes (can be found in freezer drawer 5)
- Vortexer and tube/plate spinner
- Skirted PCR plates
- 200 µl or 1.5 ml microcentrifuge tubes (having both can be useful)
- 10 μl, 20 μl, 100 µl and 200 µl pipettes and tips (multichannel pipettes are **extremely** useful)

**Procedure:**

This library preparation will take most of a day, although after the first time it shouldn’t be a long day.

This protocol is optimized for quarter volume reactions, which can take a maximum of 1000 ng of input DNA (though this can be slightly exceeded, fragmentation will become less efficient if you use too much input DNA).

It is vital to make a record of the indices used to label each sample. Every sample should use a unique combination of i5 and i7 primers, samples with the same combination cannot be pooled or sequenced on the same run. Try not to use a particular index primer too much, as we have limited volumes.

The fragmentation times have been optimized for *Triturus* samples, though they seem to work on other genera too. If you have a new class of samples you may have to re-optimize this step. Before fragmentation be careful to keep samples and reagents on the cool blocks.

Our NEB kit is stored in our NGS freezer drawer (number 10). When using reagents, try to take from tubes which are already in use – you can mark these with an X.

**Enzymatic fragmentation**

1. Collect the two yellow tubes from the NEBNext Ultra II FS kit – these are the reaction buffer and the enzyme mix. Thaw these tubes out and thoroughly vortex them to make sure any precipitate is resuspended.
2. Make a master mix consisting of 0.5 µl of enzyme mix per sample + 1.75 µl of reaction buffer per sample (for 16 samples this would be 8 µl of enzyme mix and 28 µl of reaction buffer). Vortex thoroughly and keep this master mix on the cool block.
3. Add 6.5 µl of each sample to a well of a skirted PCR plate, or a 200 µl PCR tube (keep them in a strip). Keep the samples on a cool block
4. Add 2.25 µl of the master mix to each sample, mix thoroughly by pipetting up and down
5. Transfer to a thermocycler and run the flowing program with heated lid set at 75 °C:
   1. 37 °C for 6:15 min
   2. 65 °C for 45 min
   3. Hold at 4 °C
6. If you are interested in how the fragmentation has performed, the results can be run on the Tapestation (obviously this sacrifices some product). The reaction can also be stored at -20 °C at this stage, though a small loss of yield may occur

**Adapter ligation**

1. Collect the ligation master mix and ligation enhancer from the NEBNext Ultra II FS kit and the NEB adapter from freezer drawer 3 (all three reagents are in tubes with red caps). Thaw these tubes out and thoroughly vortex them (you may need to pipette the ligation master mix up and down to ensure mixing).
2. Make a master mix consisting of 7.5 µl of ligation master mix per sample + 0.25 µl of ligation enhancer per sample (for 16 samples this would be 120 µl of ligation master mix and 4 µl of ligation enhancer) and thoroughly vortex.
3. Add 7.75 µl of this master mix to each sample (straight from the enzymatic fragmentation).
4. Make a 1:2 dilution of the NEB adapter consisting of 0.625 µl of the adapter per sample and 1.875 µl 0.1 x TE buffer per sample (for 16 samples this would be 20 µl of the adapter and 20 µl of 0.1x TE buffer) and thoroughly vortex.
5. Add 2.5 µl of the diluted NEB adapter to each sample, mix by pipetting the whole volume up and down at least 10 times.
6. Incubate in a thermocycler at 20 °C for 30 min with the heated lid off.
7. Collect the USER enzyme from freezer drawer 3 (also in a tube with a red cap), thaw it out and vortex.
8. Make a 1:4 dilution of the USER enzyme consisting of 0.75 µl of USER per sample and 2.25 µl of 0.1 x TE buffer per sample (for 16 samples this would be 12 µl of USER and 36 µl of 0.1 x TE buffer) and thoroughly vortex.
9. Add 3 µl of the diluted USER to each sample, mix by pipetting the whole volume up and down at least 10 times.
10. Incubate in a thermocycler at 37 °C for 30 min with the heated lid set at 48 °C

**Size selection**

1. Collect a tube of magnetic beads from the fridge and vortex vigorously to resuspend. Leave at room temperature for 30 minutes to allow the beads to equilibrate.
2. Make the volume for each sample up to 25 µl by adding 3 µl of 0.1 x TE buffer.
3. Add 10 µl of the beads to each sample, mix by pipetting the whole volume up and down at least 10 times, and incubate at room temperate for 5 minutes (Do not place the samples on the magnetic block at this stage).
4. Place samples on the magnetic block, once all the beads have stuck to the magnet and the solutions are clear, transfer the liquid to new wells on the PCR plate.
5. Remove the plate from the magnetic block
6. Add 5 µl of the beads to each sample (in the new wells) and mix by pipetting the whole volume up and down at least 10 times. Incubate at room temperature for 5 minutes.
7. Place samples on the magnetic block, once all the beads have stuck to the magnet and the solutions are clear, remove the liquid and discard.
8. Keeping the plate on the magnetic block, add 100 µl of 70% ethanol to each sample well so the beads are covered.
9. After 30 seconds remove the ethanol.
10. Repeat the previous two steps for a total of two washes.
11. Let the beads dry for 1-2 minutes, do not dry for so long the bead become cracked and brown.
12. Remove the plate the magnetic block, add 7 µl of 0.1 x TE buffer to the beads (pipette directly onto the beads, if necessary collect fluid from the bottom of the well and keep squirting it on the beads until they are all washed to the bottom of the well). Mix by pipetting the whole volume up and down at least 10 times and incubate at room temperate for 5 minutes.
13. Return the plate to the magnetic block. Once all the beads have stuck to the magnet and the solutions are clear, remove at least 5 µl of each sample to a new well or PCR strip. (Try to get as much volume as possible without taking any beads with the sample).

**Indexing**

1. Collect the Q5 master mix from the NEBNext Ultra II FS kit and the Illumina index primers from our freezer drawer. Allow to thaw and mix the Q5 master mix by vortexing.
2. To each sample add 6.25 µl of the Q5 master mix and 1.25 µl of both i5 and i7 index primers (making sure to record the unique combination used for each sample).
3. Mix by pipetting the whole volume up and down at least 10 times.
4. Transfer to a thermocycler and run the following program with the heated lid set to 105 °C:
   1. 98 °C for 30 sec
   2. 7* cycles of:
      1. 98 °C for 10 sec
      2. 65 °C for 75 sec
   3. 65 °C for 5 min
   4. Hold at 4 °C

**7 cycles work well for freshly extracted samples; poorer quality samples require more – 11 has been successful*

**Cleanup**

1. Add 14 µl of the magnetic beads to each sample. Mix by pipetting the whole volume up and down at least 10 times, and incubate at room temperate for 5 minutes (Do not place the samples on the magnetic block at this stage).
2. Place samples on the magnetic block, once all the beads have stuck to the magnet and the solutions are clear, remove the liquid and discard.
3. Keeping the plate on the magnetic block, add 100 µl of 70% ethanol to each sample well so the beads are covered.
4. After 30 seconds remove the ethanol.
5. Repeat the previous two steps for a total of two washes.
6. Let the beads dry for 1-2 minutes, do not dry for so long the bead become cracked and brown.
7. Remove the plate the magnetic block, add 24 µl of 0.1 x TE buffer to the beads (pipette directly onto the beads, if necessary collect fluid from the bottom of the well and keep squirting it on the beads until they are all washed to the bottom of the well). Mix by pipetting the whole volume up and down at least 10 times and incubate at room temperate for 5 minutes.
8. Return the plate to the magnetic block. Once all the beads have stuck to the magnet and the solutions are clear, collect at least 22 µl of each sample. (Try to get as much volume as possible without taking any beads with the sample).
9. The samples are now finished libraries and can be stored at -20 °C
10. The samples should be analyzed and quantified on the Tapestation or Fragment Analyzer. For the Tapestation, first perform a 1:20 dilution
