# Supplementary material for: NewtCap: An Efficient Target Capture Approach to Boost Genomic Studies in Salamandridae (True Salamanders and Newts)
Source: Ecol Evol. 2025 Aug 12;15(8):e71835. doi: 10.1002/ece3.71835 (PMC12343749; doi:10.1002/ece3.71835)
Supplement: Supplementary file 1 — Data S1: ece371835‐sup‐0001‐SupinfoS1.zip. [file ECE3-15-e71835-s001.zip › Protocol - Quick DNA Extraction_alcohol precipitation_JF V1.3.docx]

Protocol: Quicker genomic DNA extraction for sequence capture in *Triturus*

V 1.3 – James France (increased centrifuge times for steps 6 and 11)

Combined from protocols “Protocol: DNA extraction with Promega solution-based kit’’ used in the Babik lab and ‘’Genomic DNA Extraction for capture seq *Triturus*’’ used in the Shaffer lab.

**Reagents required:**

- Nuclei Lysis Solution A7943 or A7941 - Promega
- Protein Precipitation Solution A7953 or A7951 - Promega
- Proteinase K EO0491 or EO0492 - Fisher
- TE buffer 15661509 - Fisher
- Isopropanol 99%
- Ethanol 70%

**Equipment required:**

- Heater block set at 55 °C
- Centrifuge
- 1.5 ml endonuclease free low retention microcentrifuge tubes
- 200 μl or 100 μl pipettes and tips
- 50 ml falcon tubes, to hold reagents such as isopropanol and ethanol

**Procedure:**

Keep ProK in freezer until needed.

Prepare a tube of 99% isopropanol and put in the freezer (if possible this can prepared in bulk/ reused).

**Cell Lysis and RNAase Treatment**

1. Place a complete embryo or c. 10 mg of tissue (a chunk) in a 1.5 ml microcentrifuge tube containing 300 μl of Nuclei Lysis Solution and 3.0 μl Proteinase K (20 mg/ml). Mix with a brief vortex
2. Incubate at 55 °C until tissue has mostly digested. The time is quite variable, hatchlings will take 1 hour but embryos may take much longer unless their shells are broken. Vortex every 30 minutes if possible. If after 2-3 hrs. tissue is still intact, add another 3-5 μl of Proteinase K and continue incubation. The digestion can be left overnight if this is convenient.
3. When tissue is digested, vortex the tube well.

**Protein Precipitation**

1. Cool lysate to room temperature.
2. Add 100 μl Protein Precipitation Solution to the cell lysate mixture. Vortex vigorously to mix the tube contents until it turns milky white or becomes opaque (10-20 seconds) - if the solution does not turn milky white, add another 100 μl of protein precipitation solution and vortex again.
3. Centrifuge at the max speed (10-15 krpm) for 4 minutes. If a solid pellet has formed and there is no floating material proceed to the next step. If the pellet is gelatinous or there is still floating material in the supernatant more centrifugation may be required – up to 15 minutes for some samples.
4. Carefully pour off or pipette out the supernatant (which contains the DNA) into a new 1.5 ml microcentrifuge tube. Try to avoid transferring any solids.

**DNA Precipitation**

1. Add 300 μl 100% cold isopropanol (from freezer). Mix by inverting the tube 50 times (a rack of tubes can be inverted by placing another rack on top and holding firmly). Precipitated DNA may become visible as white strands.
2. Centrifuge at the max speed (10-15 krpm) for 4 minutes.
3. Carefully remove isopropanol (precipitated DNA should be visible as white pellet). ***Caution!*** *Pellet may be loose and sometimes it may be necessary to remove isopropanol by pipetting.* Add 300 μl 70% ethanol and invert the tube several times to wash the pellet.
4. Centrifuge at the max speed (10-15 krpm) for 4 minutes. Pour off the supernatant. ***Caution!*** *Pellet may be slippery and loose so be careful not to pour out pellet.*
5. Optionally, any remaining alcohol can be pipetted off the pellet to decrease drying time
6. Air-dry, if alcohol has been pipetted this should take less than 1 hour, otherwise up to 2 hours. Put tubes upside down with lid open on sheet of tissue. ***Caution!*** *Be careful not to lose the pellet, if necessary lie the tubes on their side.*

**DNA Hydration and storage**

1. Add 100 μl of TE buffer (or a different volume if required) and rehydrate in a **fridge** for at least 12 hrs. ***Caution!*** *Do not freeze at this stage!*
2. Quantity and quality of extracted DNA may be checked, depending on needs, using the Dropsense or other methods
3. Store long-term at -20 or -80 °C.
