# Supplementary material for: NewtCap: An Efficient Target Capture Approach to Boost Genomic Studies in Salamandridae (True Salamanders and Newts)
Source: Ecol Evol. 2025 Aug 12;15(8):e71835. doi: 10.1002/ece3.71835 (PMC12343749; doi:10.1002/ece3.71835)
Supplement: Supplementary file 1 — Data S1: ece371835‐sup‐0001‐SupinfoS1.zip. [file ECE3-15-e71835-s001.zip › Protocol - Target enrichment for Salamandridae 1.0b.docx]

Protocol: Target Enrichment for Salamandridae Samples with MyBaits

V 5.0 kit

V 1.0b – James France (Wielstra Lab, IBL - Leiden University and Naturalis)

Based on the manuals for the V 4.0 and V 5.0 MyBaits kit, with advice from Tara Luckau (UCLA Shaffer Lab). Arbor Biosciences continues to iterate their product so some procedures may differ with new kits.

Changes from V 1.0 to strip out the Naturalis lab specific details

**Reagents required:**

- The MyBaits kit (this includes the probes stored at -80 °C, and two other boxes stored at -20 °C and 4 °C respectively)
- **Optional**: C0t-1 solution (at 6000 ng/µl) - this blocking reagent has its own preparation protocol
- Magnetic beads for cleanup of the final product (we use Macherey-Nagel NucleoMag beads, but anything similar should be fine)
- KAPA HiFi hot start master mix
- i5 and i7 primers: i5: 5’ – AAT GAT ACG GCG ACC ACC GAG A*T- 3’

i7: 5’ – CAA GCA GAA GAC GGC ATA CGA GA*T- 3’

- Freshly prepared 70-80 % ethanol
- 0.1 x TE buffer
- Ultra-pure water

**Equipment required:**

- 10 μl, 20 μl, 100 µl and 200 µl pipettes and tips
- 200 µl and 1.5 ml tubes
- Magnetic separation blocks/racks for 200 µl and 1.5 ml tubes
- Cool blocks for 200 µl and 1.5 ml tubes
- Vortexer and tube spinner
- Thermocycler
- Tapestation/Bioanalyzer or some equivalent for library quantification/analysis

**Notes:**

I highly recommend reading the manual for the MyBaits V 5.0 kit, on which this protocol is partly based.

This protocol will require at least two consecutive days due to the long incubation step. For our Salamandridae bait-set we use a 30-hour incubation, but the standard is 24 hours, and it can be less for some probe-sets (you may want to optimize this incubation time yourself, in that case I’d probably start with a longer incubation period and try shortening it).

After the incubation has finished the washing step must be performed immediately, but after this is finished the bead-bound DNA can be stored at -20 °C for a few days, so the schedule is more flexible.

Working up the bead-bound DNA (post-capture amplification, cleanup and quantification) can be done in an afternoon if necessary. We usually split the beads into two batches, so we have a backup in case the PCR under-amplifies (or over-amplifies). Usually, we amplify the first batch on the second day and then analyze the results. If the PCR performs appropriately then we use the same conditions to amplify the second batch at a later date. (This might not be necessary once you have experience with the protocol, but with some of my Master’s students, this has been quite a life-saver)

We usually pool 16 libraries together with 250 ng of DNA per library (for a total 4000 ng of DNA per capture reaction). Other groups in our institution have had success of pooling many more libraries together (up to 50), and I believe the total mass of DNA can be up to at least 8000 ng of DNA per capture reaction. To begin I would suggest trying a very large amount of DNA (maybe as much as 500-1000 ng per sample) with fewer samples, and then try to increase the number of samples you use per pool.

After pooling our libraries equimolarly we use a vacuum centrifuge to reduce the volume to 7.2 μl.

The hybridization temperature can vary from 60-65°C. We use 62°C, but I suspect the smart thing is to start at 65°C and then gradually lower it if you are getting low yields. Lower temperatures give higher final yields but result in more off-target reads.

We use C0t-1 DNA from *Triturus* newts as a blocking reagent. This is basically DNA selectively enriched in repetitive sequences. We make this ourselves as apparently blocking repetitive sequences is important in getting good performance in target capture in organisms with very large genomes (*Triturus* is c.a. 30 Gb). We use C0t-1 in place of Block O and Block C from the MyBaits kit (I believe block C is human C0t-1 DNA).

For the post-capture amplification you ideally want to use as few PCR cycles as possible, since more PCR cycles will result in more useless duplicate reads. However, you obviously need enough DNA to sequence so this requires some optimization. I would recommend starting with 14 cycles, but this can be reduced as low as 8 cycles if you are still getting high enough yields.

Typically, we sequence at about 1 Gb per sample (so 16 Gb per pool). In a good run 50 % of our reads will be “on target” and 50 % of these will be PCR duplicates. This is more than enough to get good (at least 50x) coverage across most of our c.a. 7000 target sequences. Obviously, the required quantity and quality of sequencing will be very different with a different organism, probe-set and number of targets.

It's worth noting that if you have insufficient “on target” reads, you can just sequence more deeply to compensate (at a financial cost, to be sure). This doesn’t really work with PCR duplicates, after a certain point you just keep sequencing more clones of the same molecules.

Take care to keep the RNA baits at -80 °C and limit the number of freeze-thaw cycles they are exposed to. Aliquot each tube into individual 5.5 μl batches. I’ve found you can usually get 9 aliquots out of each tube (which gives you 6 bonus reactions in a 48 rxn MyBaits kit!).

**Day 1: Hybridization Setup**

*Start at least 1 hour before you plan to begin the hybridization reaction*

The idea here is that we prepare our libraries for hybridization by first incubating them with a blocking buffer (which should prevent off-target interactions). After blocking for 30 minutes, we then add in a hybridization mix including the RNA baits.

1. Collect the following components:

| Hyb N (4 °C) |
| --- |
| Hyb S (4 °C) |
| Hyb D (-20 °C) |
| Hyb R (-20 °C) |
| Block X (-20 °C) |
| Baits (-80 °C)  *use only as many aliquots as you need and keep on ice* |
| C0t-1 solution (or Blocks C and O) (-20 °C) |
| Libraries for enrichment |

1. Prepare the blocking master mix:

| Component | μl per reaction |
| --- | --- |
| Block X | 0.5 |
| C0t-1* | 5.0 |
| Total | **5.5** |

**You can replace C0t-1 with 2.5 μl of Block O and 2.5 μl of Block C*

1. For each capture reaction aliquot 5 μl of the blockers master mix to a 200 μl tube. Then add 7 μl of each library pool. Label these tubes carefully (on the top and the side). *These will now be referred to as* ***Libs****.*
2. Program the thermocycler with the following with the heated lid set at 105 °C *(there are multiple steps at the hybridization temperature - this is just to let you use the thermocycler as a timer for step 9)*:

| Temperature | Time |
| --- | --- |
| 95 °C | 5 min |
| 62* °C | 23 min |
| 62* °C | 7 min |
| 62* °C | ∞ |

**Or whatever hybridization temperature you choose*

1. Place the **Libs** in the thermocycler and begin the program. While the program is running work on the following steps:
2. Prepare the hybridization master mix in a 200 μl tube (or tubes):

| Component | μl per reaction |
| --- | --- |
| Hyb N | 9.25 |
| Hyb S* | 0.5 |
| Hyb D | 3.5 |
| Hyb R | 1.25 |
| Baits | 5.5 |
| Total | **20** |

**The addition of Hyb S may cause some precipitation, this should disappear after step 8*

1. Incubate the hybridization master mix in the thermocycler for 10 minutes at the hybridization temperature (wait till it reaches this temperature), take out and vortex occasionally.
2. For each capture reaction, aliquot 18.5 μl of Hybridization Mix to a 200 μl tube. *These will now be referred to as* ***Hybs****.*
3. After the **Libs** have been incubating at the hybridization temperature for 23 minutes place the **Hybs** in the thermocycler. After another 7 minutes transfer 18 μl of each **Hyb** to a **Lib**, mix by pipetting up and down 10 times. Then close the lid for the incubation period.

**Day 2: Bind & Wash**

*Start at least 1 hour before the hybridization reaction is due to end*

To isolate our RNA-bound DNA, we need to bind it to streptavidin coasted magnetic beads and then wash repeatedly to remove all the non-target DNA.

Before we use the beads, we need to remove them from their storage buffer, and replace it with a binding buffer. We do this by repeatedly pelleting the beads with a magnet and then resuspending in the binding buffer.

The wash and binding buffers come in almost identical bottles, be careful not to mix them up.

When washing non-target DNA off the beads it’s important to keep the reaction at high temperature as much as possible, since non-target DNA may be able to bind to the baits at lower temperatures (obviously some cooling can’t be avoided, especially when pelleting the beads on the magnetic block, but try to minimize the length of time the beads stay suspended at room temperature).

1. Collect the following components:

| Wash Buffer (4 °C) |
| --- |
| Binding Buffer (4 °C) |
| Hyb S (4 °C) |
| Streptavidin Beads (4 °C) |
| Buffer E (-20 °C) |
| Ultra-pure Water |

1. Prepare diluted wash buffer:

| Component | μl per reaction |
| --- | --- |
| Wash Buffer | 200 |
| Hyb S | 8 |
| Ultra-pure water | 792 |
| Total | **1000** |

1. Divide the diluted wash buffer into 190 μl aliquots (4 per library) and place in the thermocycler at least 30 min before the end of the hybridization reaction
2. For each capture reaction add 30 μl of streptavidin coated beads to a 1.5 ml tube. Pellet the beads with the magnetic rack and then remove the supernatant
3. For each reaction add 200 μl of Binding Buffer to the beads. Vortex to resuspend and then pellet the beads with magnetic rack before removing the supernatant. Repeat this step for a total of 3 cycles.
4. Resuspend in 70 μl of Binding Buffer per reaction. Transfer each aliquot to 200 μl tubes.
5. Once the hybridization reaction has finished, keep the thermocycler at the hybridization temperature and place the bead aliquot tubes inside for 2 minutes.
6. Transfer each capture reaction to the heated bead aliquots. Mix by pipetting then allow the libraries to incubate with the beads for 5 minutes (gently agitate halfway through).
7. Remove the beads from the thermocycler (keep it at the hybridization temperature) and place on the magnetic block. Once the beads form a pellet remove the supernatant. *It may be wise to store this supernatant, since any target DNA not bound to the beads will be here.*
8. Remove the samples from the block and resuspend the beads in 180 μl diluted wash buffer. Mix by pipetting then place in the thermocycler for 5 minutes (gently agitate halfway through).
9. Repeat steps 9 and 10 for a total of 4 washes
10. After the final wash, pellet the beads, remove the supernatant and resuspend in 30 μl Buffer E.
11. **Optionally** Split each pool into two batches of 15 μl, label them A and B.
12. The bead bound libraries can now be stored at -20 °C for short periods. *Usually, we proceed immediately to the amplification with the A pools and save the B pools for a later day.*

**Day 2/3: Amplification & Cleanup**

***If you choose to split the pools into A and B batches****: Perform the amplification/cleanup on the A pool first and check the results on the Tapestation/Bioanalyzer. If the results are good, then repeat the procedure exactly with the B pool. If the results from the A pool are suboptimal then change the PCR program to compensate.*

After the PCR reaction is fully prepared, we split the 50 μl into 4 tubes (this is weird, but according to the Shaffer Lab’s protocol this reduces PCR duplicates, I’m not sure it does, but it’s a zero effort step). These are recombined into one tube as soon as the PCR reaction is finished.

The presence of the streptavidin beads does not interfere with the PCR as long as the KAPA HiFi Master Mix is used **(if you use another polymerase, you might have to remove the beads before the PCR).**

In step 4 you discard beads and **keep the supernatant**. In step 6 you **keep the beads** and discard the supernatant. This is easily confused.

1. Prepare the following PCR mix:

| Component | μl per reaction |
| --- | --- |
| Ultra-pure water | 5 |
| 2x KAPA HiFi Hot Start Master Mix | 25 |
| i5 primer (10 μM) | 2.5 |
| i7 primer (10 μM) | 2.5 |
| Bead bound library | 15 |
| Total | **50** |

1. **Optionally** Split the reaction into four 12.5 μl aliquots in 200 μl tubes. Place the aliquots in the thermocycler with the following program (set the heated lid to 105 °C):

| Temperature* | Time* |  |
| --- | --- | --- |
| 98 °C | 3½ min (210 sec) |  |
| 98 °C | 30 sec | 14† cycles  (Adjust to yield) |
| 58 °C | 30 sec |  |
| 70 °C | 45 sec |  |
| 72 °C | 5 min |  |
| 4 °C | ∞ |  |

**I changed this PCR quite a lot from the MyBaits manual, this has resulted in significantly fewer PCR duplicates*

*†Or whatever number of cycles you choose to optimize around*

1. **If you performed step 4** Resuspend the beads and combine the 4 aliquots back into 1 tube
2. Pellet the beads on the magnetic block. Carefully transfer the **supernatant** to a new tube and discard the beads.

***Cleanup***

1. Add 90 µl of the magnetic beads (**Normal size-selection beads! not from the MyBaits kit**) to each sample. Mix by pipetting the whole volume up and down at least 10 times, and incubate at room temperate for 5 minutes.
2. Place samples on the magnetic block and pellet the beads. Discard the supernatant.
3. Keeping the samples on the magnetic block, add 180 µl of 70-80 % ethanol to each sample well so the beads are covered. After 30 seconds remove the ethanol and repeat for a total of 2 washes.
4. Let the beads dry for 1-2 minutes, do not dry for so long the beads become cracked and brown.
5. Remove the samples from the magnetic block, add 24 µl of 0.1 x TE buffer to the beads Mix by pipetting the whole volume up and down at least 10 times and incubate at room temperate for 5 minutes.
6. Return the samples to the magnetic block. Once all the beads have stuck to the magnet and the solutions are clear, collect at least 22 µl of each sample. (Try to get as much volume as possible without taking any beads with the sample).
7. The finished pools can be stored in a 1.5 ml tube at -20 °C indefinitely.

***Quantification and Pooling***

1. The samples should be analyzed and quantified using a Tapestation/Bioanalyzer; In our lab the target yield from the amplification is 5-15 nM of DNA in 20 μl.
2. **If you split the pools into A and B batches:** Assuming the A pool gives a good yield, begin work on the B Pool (probably on another day) with the same settings. If the A pool is not optimal then change the number of PCR cycles to increase/decrease the yield (see below).
3. **If you split the pools into A and B batches:** When the B pool is done, quantify it on the Tapestation/Bioanalyzer too. If both A and B have good yields then they can be pooled together for sequencing. Equimolar pooling is preferable here, since that will maximize the number of unique reads.

**Final Notes:**

As mentioned, in our lab the target yield from the amplification is about 5-15 nM of DNA in 20 μl. This will obviously vary depending on specific requirements. For example, our sequencing partner requires a minimum concentration of 5 nM and volume of 20 μl for NGS samples.

It’s worth noting that it is also possible for the yield to be *too high*. Generally, this indicates that the percentage of reads “on-target” will be lower than desired. This can be due to insufficient blocking, too low a hybridization temperature or mistakes in the washing step (which is surprisingly finnicky).

With the split pool system. If the A pool results in a below target concentration, we will amplify the B pool with more cycles (15 or 16 depending on how low the A concentration was). If the yield of A is too high the B pool will be amplified with just 12 or 13 cycles (fewer PCR duplicates is always good). We may also pay to sequence more deeply to compensate.
