# Supplementary material for: NewtCap: An Efficient Target Capture Approach to Boost Genomic Studies in Salamandridae (True Salamanders and Newts)
Source: Ecol Evol. 2025 Aug 12;15(8):e71835. doi: 10.1002/ece3.71835 (PMC12343749; doi:10.1002/ece3.71835)
Supplement: Supplementary file 1 — Data S1: ece371835‐sup‐0001‐SupinfoS1.zip. [file ECE3-15-e71835-s001.zip › sm_0001-FigS1-S2.docx]

**Supplementary Figures**

**
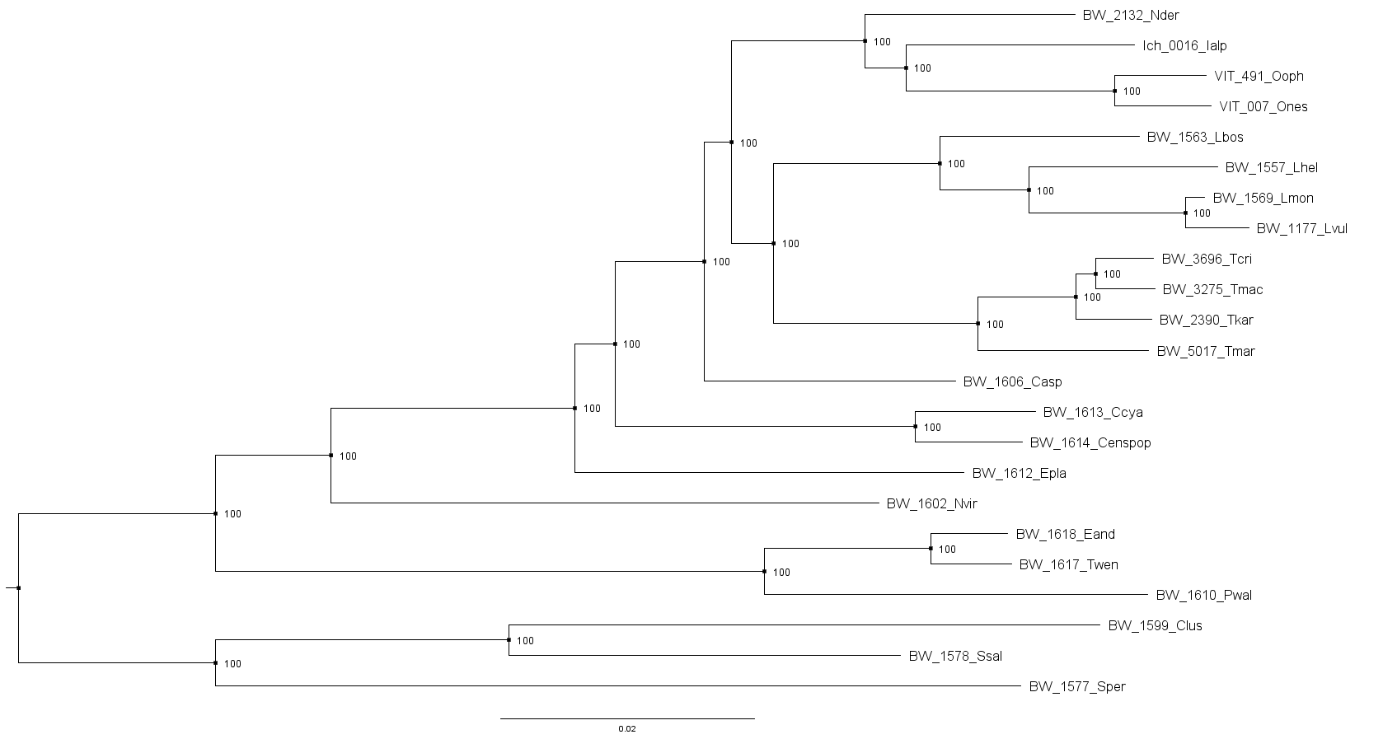

*Figure S1: The raw, reconstructed NewtCap-based phylogeny of the Salamandridae family.*** *This is the same tree as provided in MS Fig. 1, but with original sample identifiers and bootstrap values.* *The tree is rooted on the branch separating the newts and the clade containing the true salamanders and* Salamandrina *(see also Fig. S2, which confirms the root position adopted here).*

**
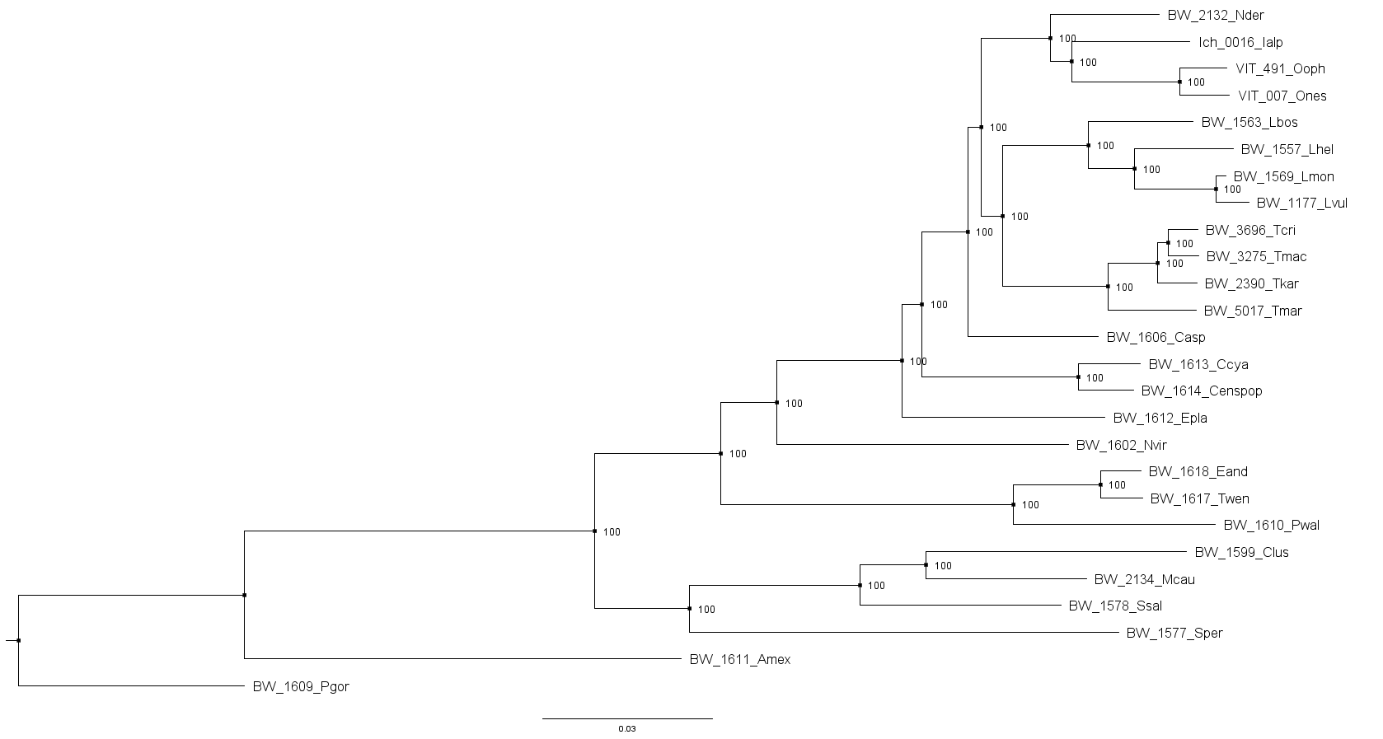

*Figure S2: The raw, reconstructed NewtCap-based phylogeny of the Salamandridae family, including two non-salamandrids.*** *This tree is the result of an independent RAxML analysis that also includes a* Mertensiella caucasica *individual, and two non-salamandrid individuals: one* Ambystoma mexicanum *sample and one* Paradactylodon gorganensis *sample. The tree is based on 265,105 SNPs and shows sample identifiers and bootstrap values.* *The tree is rooted on the branch of* P. gorganensis*, which belongs to the Hynobiidae family and is more distantly related to Salamandridae than* A. mexicanum*, which belongs to the Ambystomatidae family (Marjanović and Laurin, 2013, Frost, 1985).*
