# Supplementary figures and images for: NewtCap: An Efficient Target Capture Approach to Boost Genomic Studies in Salamandridae (True Salamanders and Newts)
Source: Ecol Evol. 2025 Aug 12;15(8):e71835. doi: 10.1002/ece3.71835 (PMC12343749; doi:10.1002/ece3.71835)

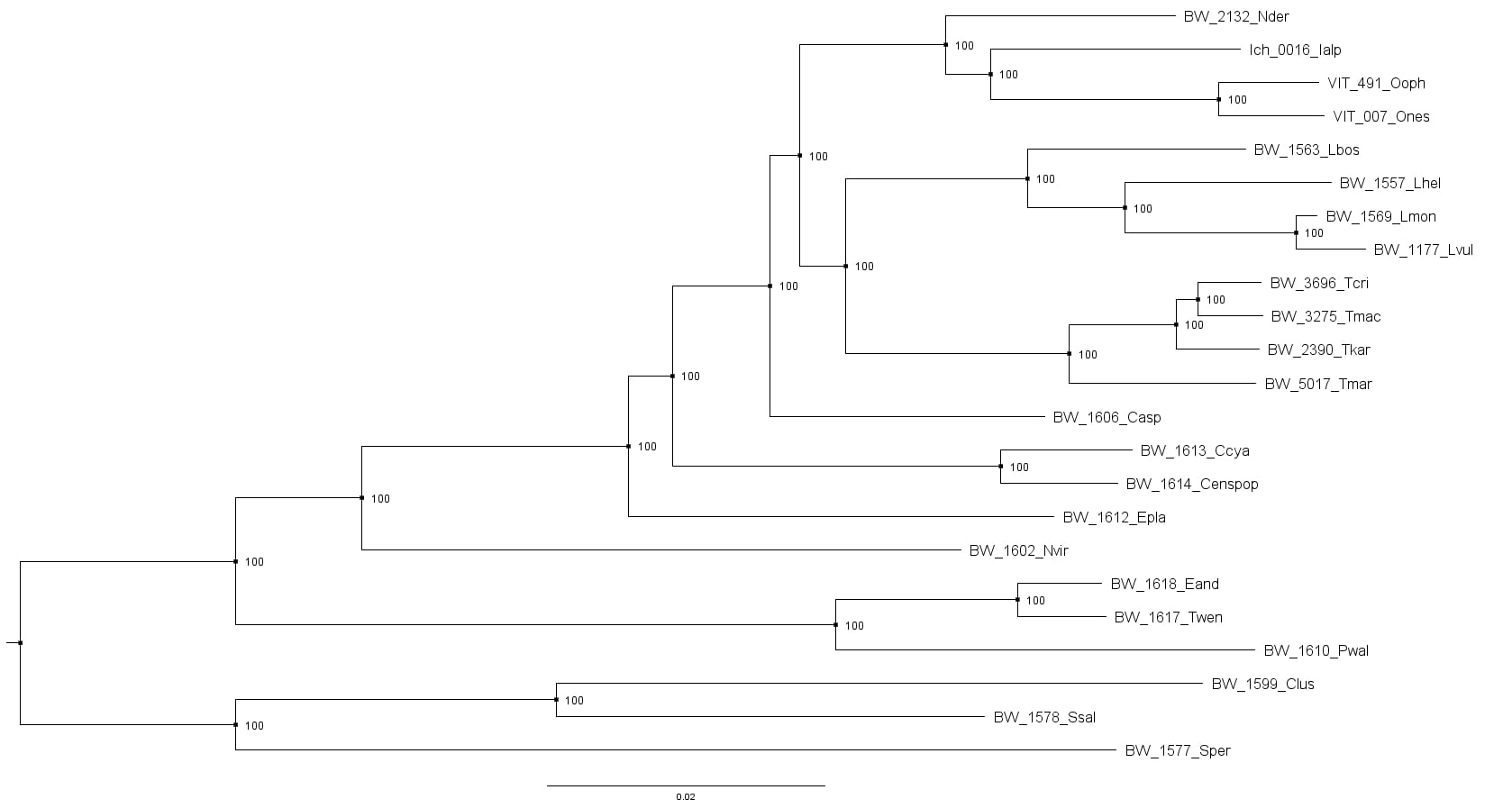

Supplement: Supplementary file 1 — Data S1: ece371835‐sup‐0001‐SupinfoS1.zip. [file ECE3-15-e71835-s001.zip › Fig_S1.jpg]

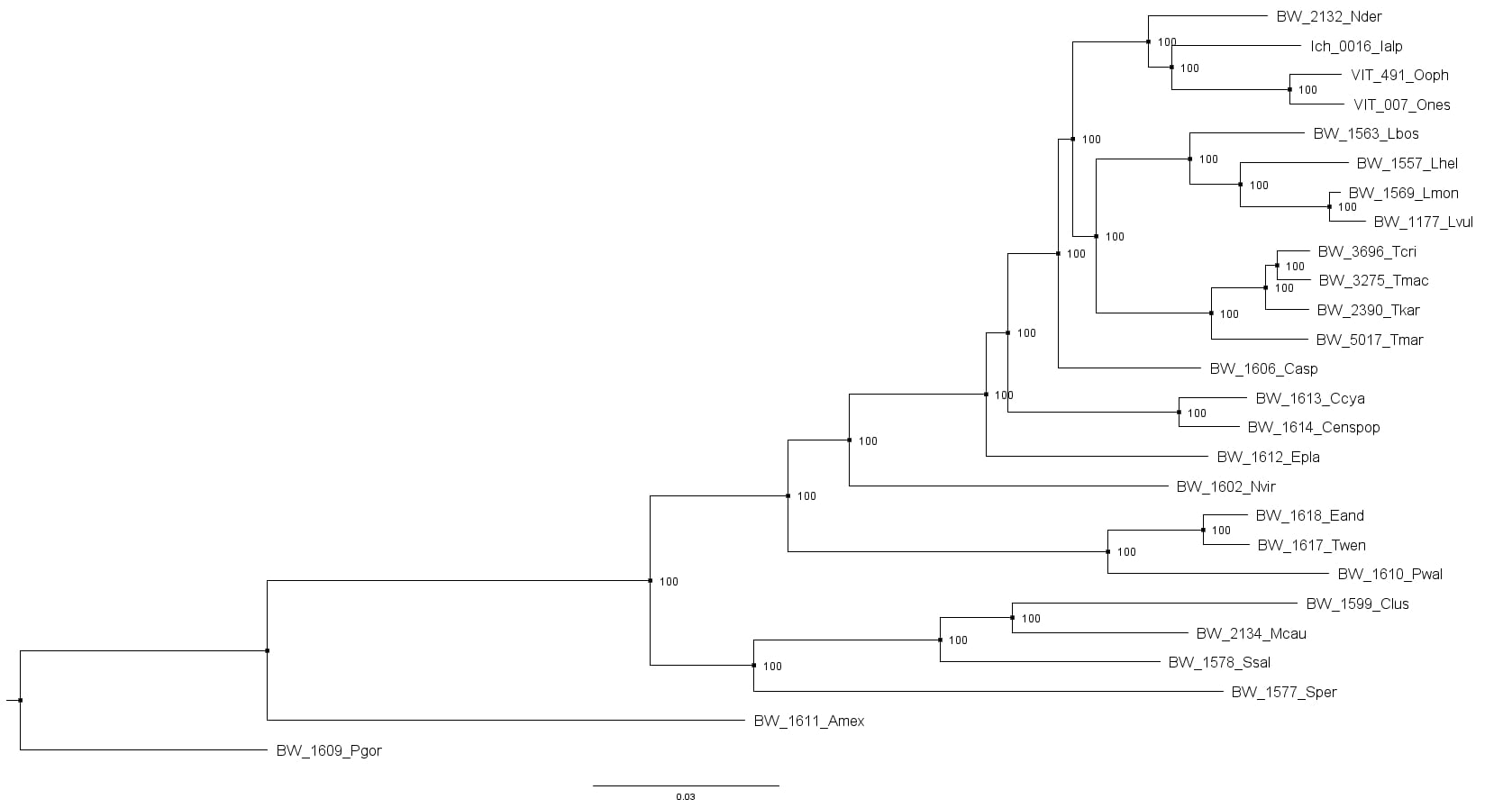

Supplement: Supplementary file 1 — Data S1: ece371835‐sup‐0001‐SupinfoS1.zip. [file ECE3-15-e71835-s001.zip › Fig_S2.jpg]
